# Single Cell Transcriptomics Reveal Abnormalities in Neurosensory Patterning of the *Chd7* Mutant Mouse Ear

**DOI:** 10.3389/fgene.2018.00473

**Published:** 2018-10-23

**Authors:** Robert Durruthy-Durruthy, Ethan D. Sperry, Margot E. Bowen, Laura D. Attardi, Stefan Heller, Donna M. Martin

**Affiliations:** ^1^Departments of Otolaryngology – Head and Neck Surgery, Stanford University, Stanford, CA, United States; ^2^Medical Scientist Training Program, University of Michigan, Ann Arbor, MI, United States; ^3^Department of Human Genetics, University of Michigan, Ann Arbor, MI, United States; ^4^Division of Radiation and Cancer Biology, Department of Radiation Oncology, Stanford University, Stanford, CA, United States; ^5^Department of Pediatrics and Communicable Diseases, University of Michigan, Ann Arbor, MI, United States

**Keywords:** CHD7, chromatin remodeling, inner ear development, single cell genomics, otic vesicle, mouse models, CHARGE syndrome, patterning

## Abstract

The chromatin remodeling protein CHD7 is critical for proper formation of the mammalian inner ear. Humans with heterozygous pathogenic variants in *CHD7* exhibit CHARGE syndrome, characterized by hearing loss and inner ear dysplasia, including abnormalities of the semicircular canals and Mondini malformations. *Chd7*^Gt/+^ heterozygous null mutant mice also exhibit dysplastic semicircular canals and hearing loss. Prior studies have demonstrated that reduced *Chd7* dosage in the ear disrupts expression of genes involved in morphogenesis and neurogenesis, yet the relationships between these changes in gene expression and otic patterning are not well understood. Here, we sought to define roles for CHD7 in global regulation of gene expression and patterning in the developing mouse ear. Using single-cell multiplex qRT-PCR, we analyzed expression of 192 genes in FAC sorted cells from *Pax2Cre;mT/mGFP* wild type and *Chd7*^Gt/+^ mutant microdissected mouse otocysts. We found that *Chd7* haploinsufficient otocysts exhibit a relative enrichment of cells adopting a neuroblast (vs. otic) transcriptional identity compared with wild type. Additionally, we uncovered disruptions in pro-sensory and pro-neurogenic gene expression with *Chd7* loss, including genes encoding proteins that function in Notch signaling. Our results suggest that *Chd7* is required for early cell fate decisions in the developing ear that involve highly specific aspects of otic patterning and differentiation.

## Introduction

Accurate structure-function morphogenesis in the mammalian inner ear requires precisely orchestrated events controlling cellular proliferation and differentiation. Auditory and vestibular signals are received by the inner ear and transmitted to the brain via the eighth cranial nerve. Proper function of the inner ear is fundamental for our senses of hearing and balance. Inner ear morphogenesis begins with the formation of the otic placode from the pre-placodal region locatedclose to the developing rhombencephalon (Fekete, 1996; Alsina and Whitfield, 2017). The otic placode elongates along the ectodermal ridge and invaginates to form the otic cup and later the otic vesicle, located between the ectoderm layer and the lateral wall of the neural tube. Over the next several days of development (E9.5–E15.5 in the mouse), the otic vesicle grows and segregates into the various epithelial compartments that ultimately give rise to the semicircular canals, vestibular and auditory ganglia, and sensory epithelia such as the sensory cristae and maculae, and the organ of Corti. Coordination of this complex developmental process depends on accurate spatiotemporal control of gene expression (Fekete, 1996; Fekete et al., 1997; Fekete and Wu, 2002).

Heterozygous null mutations in *CHD7*, the gene encoding chromodomain helicase DNA binding protein 7, cause CHARGE syndrome, a disorder characterized in part by global sensory deficits including malformation and dysfunction of the ear (Vissers et al., 2004). Individuals with CHARGE syndrome and *CHD7* haploinsufficiency exhibit abnormalities in development of the outer, middle, and inner ear, and highly penetrant lateral and posterior semicircular canal abnormalities manifesting in difficulties with sound capture, transduction, signal processing, and balance (Sanlaville et al., 2006; Choo et al., 2017). CHD7 functions through ATP-dependent nucleosome remodeling, which exposes or masks regions of genomic DNA to access by *trans*-acting factors that are critical for initiating or maintaining gene expression (Bouazoune and Kingston, 2012). Studies in *Chd7* haploinsufficient mice largely replicate the inner ear findings reported in humans, including hearing loss, lateral and posterior semicircular canal malformations, and vestibular innervation defects (Adams et al., 2007; Hurd et al., 2010, 2011, 2012). The effects of *Chd7* haploinsufficiency on mouse inner ear development are complex, profound, and likely result from early stage (E8.5–E10.5) disruptions of gene expression networks in the developing ear.

Loss of *Chd7* also results in major disruptions to the transcriptome in several CHARGE-relevant cell types and tissues. Microarray analysis and RNA-sequencing of *Chd7* mutant mouse embryonic stem, neural stem, and cerebellar granule precursor cells have uncovered abnormalities in expression of hundreds of genes involved in developmental signaling pathways (Engelen et al., 2011; Feng et al., 2013, 2017; Schulz et al., 2014; Whittaker et al., 2017; Yao et al., 2018). Germline loss of a single copy of *Chd7* in the developing mouse inner ear disrupts expression of transcription factors, signaling molecules, and structural proteins, illuminating the genetic basis for the broad phenotypic impact of this chromatin remodeler on ear development (Hurd et al., 2010, 2012). To date, studies of the *Chd7* mutant mouse ear have relied on analysis of individual genes using *in situ* hybridization or immunohistochemistry in tissue sections or whole embryos. However, the effects of CHD7 *in vivo* occur within a complex three-dimensional architecture marked by dynamic cellular differentiation and morphogenetic events.

The E10.5 otocyst comprises a sphere whose domains of gene expression lead to the compartment-boundary model originally proposed by Fekete (1996) as octants containing cells that behave similarly, and express related genes. These octant domains of gene expression in the developing ear are thought to behave in a similar fashion to other segmented anatomic structures, including the *Drosophila* wing and vertebrate brain, where cells occupy specific compartments that act synergistically or antagonistically to establish borders and specific identities. For example, antagonism between members of the SHH and WNT signaling pathways help specify the dorsal and ventral halves of the otocyst, while the neurosensory patches require localized TGFB and Notch signaling (Fekete, 1996; Groves and Fekete, 2012). Moreover, individual structures (e.g., endolymphatic duct, semicircular canals, cochlea) reproducibly derive from specific regions of the vertebrate otocyst, suggesting links between gene expression boundaries and structural differentiation. For example, anatomically, the ear can be divided into the dorsal and ventral compartments which give rise to the vestibular and auditory systems, respectively. Other structures in the developing ear, including the lateral semicircular canal, are generally understood to arise from lateral compartments, while neuroblasts that migrate from the otocyst to the vestibulocochlear (VIII) ganglion arise from the ventrolateral compartment and migrate medially before delamination.

Studies have shown that multiple genes direct this highly organized and complex developmental process of otic patterning. Given the broad impact of CHD7 loss on the mammalian ear, we hypothesized that CHD7 may also play important roles in early otic patterning. Here we used single-cell qRT-PCR and *in silico* tissue reconstruction to determine the effects of *Chd7* deficiency on genetic specification of the developing mouse otocyst. Our results show that CHD7 guides neuroblast production, potentially via effects on Notch signaling.

## Materials and Methods

### Mouse Husbandry

*Chd7*^Gt/+^ mice were maintained on a C57BL/6J X 129/Sv mixed background and genotyped as previously described (Adams et al., 2007). *Gt(ROSA)26Sor*^mtdTomato.mEGFP^ and *Pax2*^Cre+/-^ mice were also genotyped as previously described (Durruthy-Durruthy et al., 2014). All mouse husbandry and experimental handling was approved by and performed according to the standards of the University of Michigan and Stanford University Committees on the Use and Care of Animals (UCUCA).

### Otocyst Isolation

*Chd7*^Gt/+^ mice were crossed with *Gt(ROSA)26 Sor*^mtdTomato.mEGFP^ mice (hereafter called *mT/mGFP*) to generate *Chd7*^Gt/+^*;mT/mGFP* mice. *Pax2*^Cre+/-^ male mice were crossed with *Chd7*^Gt/+^*;mT/mGFP* mice, and females inspected daily for vaginal plugs. Embryos were collected from pregnant females by hysterectomy on E10.5 and placed in cold Hank’s balanced salt solution (HBSS). Yolk sacs and caudal aspects of the embryos were collected and processed for genotyping. Otocysts and surrounding tissues were microdissected and incubated at 37°C in thermolysin to digest the mesenchyme. Otocysts were then washed with 1 X HBSS and treated for 30 min at 37°C with Accutase (Innovative Cell Technologies, San Diego, CA, United States). Mechanical trituration was used to dissociate cells, followed by washing twice with HBSS. Cell suspensions were passed through a 35 μm strainer (BD Biosciences, San Jose, CA, United States) to eliminate cell clumps.

### Fluorescence Activated Cell Sorting (FACS)

Sytox Red (ThermoFisher Scientific, Waltham, MA, United States) was added to cell suspensions to identify dead cells. Cells were sorted with a FACSARIA II (BD Biosciences). Debris and non-cellular particles were removed. Cell doublets and multiplets were removed using two consecutive gating steps: forward-scatter height (FSC-H) vs. forward-scatter area (FSC-A) and side-scatter area (SSC-A) vs. side–scatter width (SSC-W). Dead and compromised cells were identified by Sytox Red uptake and eliminated based on SSC-A vs. Sytox Red sorting. mGFP + /tdTomato-negative cells were collected into individual wells of 96-well PCR plates (USA Scientific, Ocala, FL, United States) pre-filled with CellsDirect 2 X Reaction mix (Invitrogen) and 0.05 U of SUPERase-In RNase Inhibitor (Invitrogen, Carlsbad, CA, United States). Flow rates were held at 300 cells/s (“precision” set to “single cell”; nozzle; 100 μm). Plates containing cells were sealed and stored at -80°C until RNA isolation.

### RNA Isolation and qRT-PCR

Complementary DNA (cDNA) was generated by reverse transcription with SuperScript III RT Platinum Taq Mix using primers validated by amplicon-specific DELTAgene Assays (pooled, 500 nM each; Supplementary Table S1). Reverse transcription, followed by a 20-cycles pre-amplification of target genes, was done in a thermocycler (Bio-Rad S1000). Samples were then treated with exonuclease I (NEB), diluted fivefold for subsequent PCR reactions and added to pre-mixed 2X SsoFast EvaGreen Supermix with Low ROX (Bio-Rad, Hercules, CA, United States) and 20X DNA Binding Dye Sample Loading Reagent (Fluidigm, South San Francisco, CA, United States). Assay mix contained 2X Assay Loading Reagent (Fluidigm), 1X DNA suspension buffer, and primer pairs. The 96.96 dynamic array integrated fluid circuit (IFC, Fluidigm) was primed with control line fluid and loaded using the HX IFC controller (Fluidigm). QPCRs were run on a Biomark HD (Fluidigm) for 30 cycles with generation of melting curves. For all 192 amplicons, forward and reverse primers are listed in Supplementary Table S1.

### Data Analysis

Data was processed as previously described (Durruthy-Durruthy et al., 2014). In short, *C*_t_-values for each reaction were compared to the limit of detection (LOD) *C*_t_-value. The LOD-*C*_t_-value represents the average sensitivity of the collection of DELTAgene Assays and is calculated based on the highest *C*_t_-value at which variation between technical replicates is low (LOD *C*_t_ = 23). *C*_t_-values greater that or equal to the calculated LOD *C*_t_ were treated as negative and set to zero (e.g., undetectable). Cells with GAPDH/ACTB *C*_t_-values lower than 3x standard deviation (‘compromised’ cell) or values 3x higher than standard deviation (‘multiplet’ cell) were removed from downstream analysis. Raw *C*_t_-values were transformed into Log2Ex values by subtracting it them from the LOD Ct. Data was normalized using the median Log2Ex method, as follows: for every cell, the median Log2Ex value across all genes was calculated, and the difference between this cell-characteristic value and the mean of all median Log2Ex values was subtracted from all Log2Ex values. Expression values were scaled between 0 and 1 to remove embryo-specific gene expression features (e.g., dynamic range) and to allow for comparision and differential gene expression analysis: x – min(x)/(max(x) – min(x)). This range standardization transforms all variables between 0 and 1 and retains the rank order as well as the relative size of separation between values. Downstream analysis was performed using R version 3.4.2. Two-dimensional principal component analysis (PCA) was carried out using the FactoMineR package. Three-dimensional PCA was done as outlined (Durruthy-Durruthy et al., 2015). Hierarchical clustering was performed using the heatmap.2 package. Unpaired *t*-test (unequal variance) was used to assess statisitcal significance (e.g., *p* < 0.05).

## Results

### Single-Cell Transcriptional Profiling of *Chd7* Mutant Microdissected Otocysts

Selective identification by FACS of E10.5 otocyst cells has been previously described (Durruthy-Durruthy et al., 2014, 2015). Since the developing otocyst exhibits ubiquitous and specific expression of the transcription factor PAX2, we crossed *Pax2-Cre* male mice with female mice harboring both the *Chd7*^Gt^ and two-color fluorescent reporter *ROSA^mT/mG^* alleles. From this cross, we generated wild type and *Chd7* heterozygous E10.5 embryos that selectively expressed EGFP in the developing ear, allowing specific microdissection and FACS of 479 otic cells. The cells were collected over three different litters from individual embryos (three wild type, four *Chd7*^Gt/+^), with no significant differences in cell size, cell viability, intracellular granularity or complexity across five assay plates, litters, or genotypes (Supplementary Figure S1A).

The 479 otic cells were subjected to microfluidic qRT-PCR for 192 transcripts (Supplementary Table S1), including 88 transcripts queried in a previous analysis of wild type mouse otocyst cells (Durruthy-Durruthy et al., 2014). Of 479 total cells, 13 failed quality control and were excluded from subsequent analysis, leaving 215 *Chd7*^+/+^ and 251 *Chd7*^Gt/+^ cells (Supplementary Figure S1A). Principal component analysis and hierarchical clustering revealed no differences among the cells based on embryo (Supplementary Figure S1B) or assay plate (Supplementary Figure S1C). Hierarchical clustering corroborated this finding (Supplementary Figure S1D). The lack of batch effects shown by these analyses confirmed that there were no differences in the integrity or quality of cells between embryos or plates to confound further analysis.

### Loss of *Chd7* Results in Relative Enrichment of Neuroblasts vs. Otic-Derived Epithelial Cells

**FIGURE 1:**
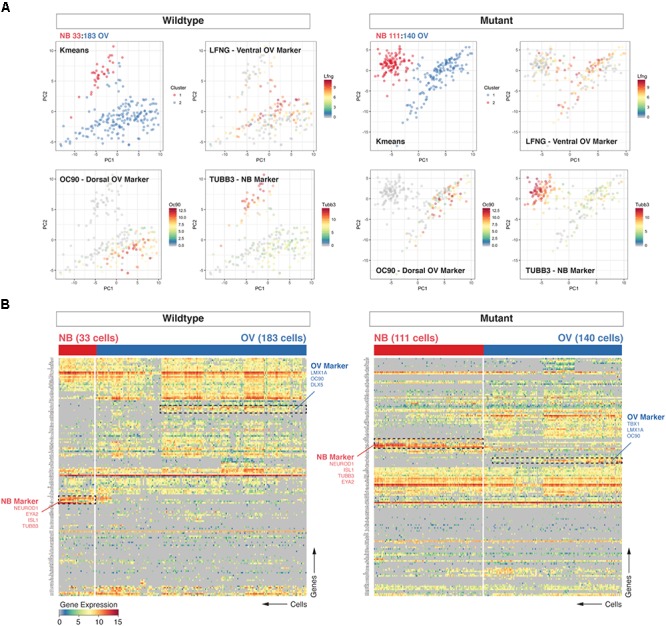
Loss of *Chd7* shifts the distribution of cells the E10.5 otocyst toward those with neuroblast identity. Principal component analyses (PCA) **(A)** and hierarchical clustering **(B)** on *Chd7*^+/+^ and *Chd7*^Gt/+^ E10.5 otic derived cells distinguishes otic epithelia cells from putative neuroblast cells and reveals a relative increase in the proportion of *Chd7*^Gt/+^ cells expressing pro-neural genes compared to wild type. Cells projected onto first two components are color-coded based on k-means cluster and expression levels of three representative markers (*Lfng* = ventral otic epithelium, *Oc90* = dorsal otic epithelium, *Tubb3* = delaminated neuroblasts). This was confirmed by increases in pro-neural (*Neurod1, Tubb3*) vs. pro-epithelial (*Oc90*) gene expression amongst *Chd7* heterozygous cells.

Upon morphogenesis of the otic placode into the otocyst, a proportion of cells beginning at E8.5 undergoes differentiation along the anteromedial aspect into neuroblasts, which then delaminate and migrate to populate the nascent vestibulocochlear ganglion (Liu et al., 2000; Kim et al., 2001; Fekete and Wu, 2002). We used cluster analysis (hierarchical clustering and k-means) to group cell populations and found that the two most prominent clusters discriminate cells of neuroblast identity (e.g., expressing *Neurod1, Tubb3*) from otic epithelial associated cells (e.g., expressing dorsal marker *Oc90* or ventral marker *Lfng*) cells (Durruthy-Durruthy et al., 2014). Principal component analysis corroborated the partitioning of the data into those two distinct groups of cells (Figures 1A,B). We further found an interesting discrepancy between the two data sets in the relative proportion of neuroblasts from *Chd7*^Gt/+^ (111/251 or 44.2%) *vs.* wild type (33/216 or 15.3%) cells. Calculating the fraction of neuroblast cells on a per-embryo basis confirmed this finding and showed the difference between mutant and wildtype samples to be significant (unpaired *t*-test, *p* = 0.0095, Supplementary Figure S2).

### Expression of Neurogenic, but Not Dorsal Marker Genes Are Disrupted in *Chd7* Mutant Otocysts

Structures emerging from the developing otocyst are spatially and genetically compartmentalized across the three embryologic axes. The dorsal-ventral axis, generated by SHH and WNT antagonism, segregates the majority of the developing vestibular system away from the nascent auditory system (Riccomagno et al., 2002, 2005; Bok et al., 2005, 2007; Ohyama et al., 2006; Brown and Epstein, 2011). Additional structures, including the lateral semicircular canal and endolymphatic duct, arise from specific dorsal regions segmented by the medial-lateral and anterior-posterior axes, respectively. This patterning is driven by signaling factors of the TGF-beta and FGF superfamilies (Wu and Oh, 1996; Kwak et al., 2002; Hammond and Whitfield, 2011; Groves and Fekete, 2012; Ohta et al., 2016). Concordant with the three embryologic axes, eight octants have been defined that delineate the structural and genetic compartmentalization of the three-dimensional otocyst: ADL, anterodorsolateral; PDL, posterodorsolateral; PDM, posterodorsomedial; ADM, anterodorsomedial; AVM, anteroventromedial; PVM, posteroventromedial; PVL, posteroventromedial; PVL, posteroventrolateral; AVL, anteroventrolateral (Fekete, 1996; Durruthy-Durruthy et al., 2014).

Consistent with the compartment-boundary model of ear development, we hypothesized that reductions in *Chd7* dosage that lead to semicircular canal abnormalities might be a result of altered distribution of otic epithelial cells expressing dorsal-associated transcripts or, more simply, a decrease of the fraction of cells that adopt a dorsal identity. Two vestibular structures are reliably malformed in *Chd7* mutant mice: the posterior and lateral semicircular canals (Adams et al., 2007; Hurd et al., 2011). Additionally, the posterior cristae often exhibit reductions in the number of nerve calyces, with associated abnormalities in the ultrastructure of the sensory epithelium (Adams et al., 2007). As defined in Fekete and Wu (2002), the posterior semicircular canals and cristae derive from cells located in the posterior dorsolateral quadrant, while the lateral semicircular canal and crista derive from the boundary region defined by the posterior ventrolateral and posterior dorsolateral quadrants (Oh et al., 1996; Wu et al., 1996; Fekete and Wu, 2002).

**FIGURE 2:**
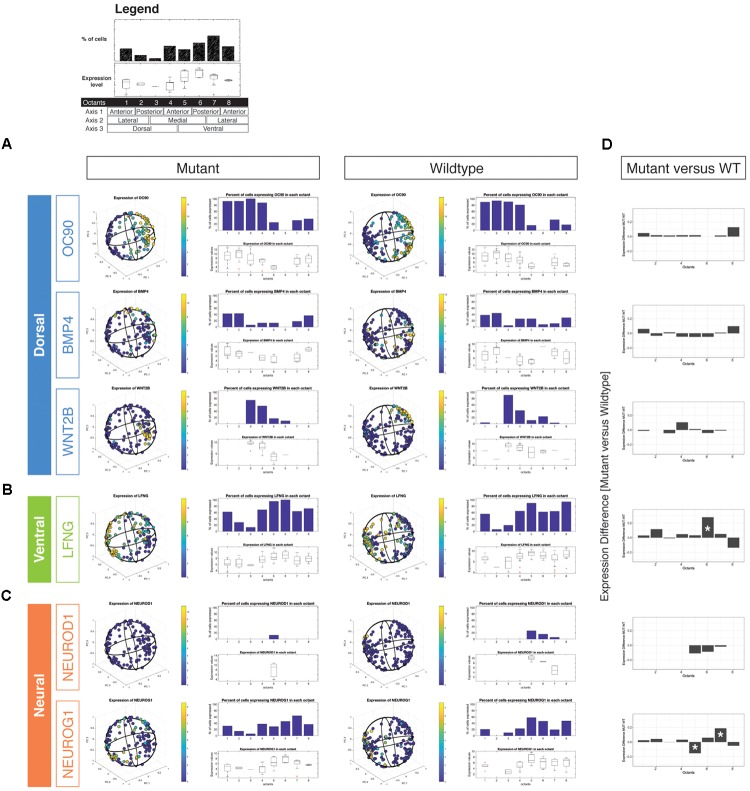
Three-dimensional reconstruction of epithelial cells from *Chd7* wild type and mutant otocysts. Octant-by-octant analysis of dorsalizing vs. ventralizing genes was performed in both the *Chd7* wild type (left) and mutant (right) populations. Dorsal genes **(A)** *Oc90, Wnt2b*, and *Bmp4* were relatively similar across wild type and mutant cell populations. In contrast, ventral gene **(B)** *Lfng* and neural genes **(C)** *Neurod1* and *Neurog1* exhibited changes in the proportion of, and amount of expression among, mutant cells across the otocyst. The legend shown on top outlines the octant-based quantification of the *in silico* otocyst model. Each number refers to an octant of the sphere (= 1/4th of on half) and is associated to a specific anatomical domain (e.g., dorso-anterio-lateral = octant 1). The bar graph shows the percentage of cells that express a gene above detection level. Box plots show gene expression levels on a per-octant basis. Differential octant-specific gene expression analysis between mutant and wild type is shown in **(D)**.

We examined wild type and *Chd7* mutant cell populations for changes in expression of the dorsal markers *Oc90, Bmp4*, and *Wnt2b* (Figure 2A). In cells classified as arising from dorsal octants (1–4), there were no significant changes in either the proportion of cells expressing dorsal markers or the aggregate expression levels of these transcripts (Figure 2D). Interestingly, there was an increase in the proportion of dorsal otic epithelial cells expressing the ventral-associated transcripts *Lfng* and *Neurog1* (Figures 2B–D). Strikingly, we also noted an increased proportion of octant 1 (endolymphatic duct) and octant 2 (posterior semicircular canal) cells expressing both *Lfng* and *Neurog1.* The results suggest that dorsally fated cells in the *Chd7* mutant may aberrantly adopt mixed dorsal and neurogenic fates.

### Subtle but Distinct Changes in the Ventral Otocyst Upon Loss of *Chd7*

In mice, loss of *Chd7* results in subtle but damaging defects to the ventral aspect of the developing ear. Low-power microscopic examination of the *Chd7*^Gt/+^ saccule, utricle, and cochlea reveals no significant anatomic dysmorphology; however, *Chd7*^Gt/+^ mice exhibit mixed conductive-sensorineural hearing loss (Hurd et al., 2010, 2011). *Chd7* is expressed in mature inner and outer hair cells, as well as in spiral ganglion neurons, yet no notable ultrastructural defects in these cell types have been uncovered (Adams et al., 2007; Hurd et al., 2010). It is also not known whether persistence of *Chd7* expression in hair cells and spiral ganglion neurons at later stages (i.e., beyond development) is necessary for proper auditory function. Given the high expression of *Chd7* in the otocyst (Hurd et al., 2010), we hypothesized that *Chd7* may function early to promote specification or patterning of the developing otocyst.

By E10.5, cells occupying the ventral otic epithelial compartment express high levels of *Lfng* with low-moderate transcription of *Neurod1*, and *Neurog1* (Figures 2B,C). We did not find massive shifts in expression of these genes between mutant and wildtype reconstructed otocysts, but we noted subtle differences. *Neurod1* expression was reduced in mutant otocysts and restricted to a small subset of cells associated with octant 5, which has anterior ventro-medial (AVM) identity. *Neurog1* expression, in contrast, appeared to be found more broadly across ventral and also dorsal compartments, compared with wild type. A significant reduction of *Neurog1* expression was noted in octant 5 (AVM) whereas more cells in octant 7 (PVL) expressed the gene (Figures 2C,D). Misexpression of neurogenic genes in cells from these *Chd7*^Gt/+^ ventral octants correlates with compromised epithelial development of the lateral semicircular canal, saccule, and cochlea (Wu and Oh, 1996).

### Notch Gene Expression Is Expanded Upon Loss of *Chd7*

**FIGURE 3:**
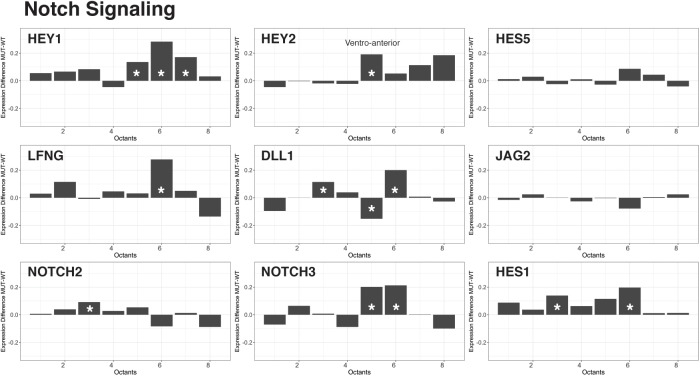
Effectors of Notch signaling are upregulated in the *Chd7*^Gt/+^ ventral otocyst. Analysis of Notch effector gene transcription across cells from all eight octants revealed an increase in *Hey1, Hey2, Lfng, Dll1, Notch3*, and *Hes1* transcription in ventral octants 5–8 in *Chd7^Gt/+^* vs. wild type otic vesicle cells.

As described above, several critical signaling pathways, including TGF-beta, FGF, and Notch, operate during early inner ear development (Groves and Fekete, 2012; Galvez et al., 2017). We compared the reconstructed and spatially defined *Chd7*^+/+^ and *Chd7*^G/+^ cell populations to uncover global changes in genes belonging to these signaling factor superfamilies that drive otic patterning. We found that in *Chd7*^Gt/+^ otic epithelial cells, *Hey1* and *Hey2* were upregulated in ventral octants 5–8 (AVM, PVM, PVL) (Figure 3). Both *Hey1* and *Hey2* reside in the Notch pathway and have been implicated in pro-neurosensory cell specification along the ventro-anterior aspect of the developing otocyst (Adam et al., 1998; Eddison et al., 2000; Neves et al., 2011, 2013). Together with *Hes1*, these Notch effectors have critical roles in limiting neural precursor cell differentiation into neurons (Sakamoto et al., 2003). From our data, loss of *Chd7* appears to derestrict expression of *Hey1* and *Hey2*. These changes are accompanied by additional differences in expression of Notch signaling related genes mainly in the ventral compartments of the otocyst. We interpret these data as an indication that expression changes of Notch signaling pathway components in the ventral part of the otocyst of *Chd7*^Gt/+^ mice ultimately lead to changes in neuroblast numbers at E10.5.

## Discussion

In this manuscript, we show that single copy loss of *Chd7*, the gene encoding chromodomain helicase DNA binding protein 7, leads to changes in gene expression and otic cell patterning in single cells derived from the developing mouse embryo. Single cell analysis using multiplexed qRT-PCR of 192 genes revealed a significant shift in the distribution of otic-derived cells vs. neuroblasts in Pax2^Cre+/-^; Chd7^Gt/+^;mT/mGFP mice compared to Pax2^Cre+/-^; Chd7^+/+^; mT/mGFP littermates. We found that loss of Chd7 also leads to enrichment of proneural and Notch-related gene expression in cells that map to the ventral compartments of the otocyst, with minimal disruption of gene expression in dorsal-derived cells. This study is the first to apply single cell analysis to mutant mouse inner ear tissues, and highlights the power and limitations of this approach to dissect molecular events associated with complex pathologies.

Comparison of our results to previous studies sheds light on potentially novel roles for CHD7 in cell proliferation and/or specification of prosensory cells and neuroblasts in the inner ear. Earlier work from our laboratory showed, by immunostaining with early neuronal markers, that loss of one or two copies of *Chd7* in the developing mouse ear results in transiently fewer neuroblasts in the E10.5 with recovery by E11.5 (Hurd et al., 2010). In contrast, here we found that heterozygous loss of *Chd7* leads to enrichment of developing neuroblasts. There are a number of possible explanations for this discrepancy. First, the single cell results presented here were performed only at E10.5, and do not capture dynamic changes in neuronal markers. Thus, it is possible that at the level of mRNA, neuroblastogenic transcription is increased with loss of *Chd7*, whereas protein levels are reduced. Second, there may be differences in genetic background between this study and our earlier report, since breeding of the *Chd7*^Gt/+^ mice with *Pax2Cre*^+/-^ and *mT/mGFP* reporter mice may have introduced additional genetic factors which could modify developmental phenotypes. Third, loss of *Pax2* (in *Pax2Cre*^+/-^ mice) may influence *Chd7* deficiency effects on inner ear neuroblasts, and the *Pax2Cre*-mediated mT/mGFP reporter may label a subset of cells that are somehow preferentially fated to become neuroblasts. Finally, the observed phenotype might be the result of a delayed neuroblast delamination, resulting in a slowdown of neuroblast migration and thereby leading to an apparent discrepancy. The observation that discrepancies in neuroblast cell numbers at E10.5 are transient supports this interpretation. The observed effects therefore could be the result of differences in spatial distribution of not yet delaminated, migrating neuroblasts, and/or of neuroblasts that already arrived at the location of the developing cochleo-vestibular ganglion. Tissue dissection at E10.5 mainly yields neuroblasts in or near the otocyst. Regardless of these caveats, however, our comparisons relied on littermate embryos and we observed no major variability in effects between litters, which should minimize the influence of both different genetic backgrounds and *Pax2* deficiency.

The exact role of *Chd7* in specification of prosensory cells vs. neuroblasts remains to be determined. *Chd7* is highly expressed in the developing otocyst and surrounding mesenchyme (Hurd et al., 2010), and in the nascent cochleovestibular ganglion as early as E9.5. *Chd7* is also expressed in sensory auditory and vestibular epithelia, where it becomes enriched in hair cells and has lower expression in supporting cells. This expression pattern suggests that *Chd7* regulates proneurosensory cell fate (sensory epithelium vs. neuroblast), perhaps at the expense of other cell types. The increased proportion of neuroblasts in *Chd7*^Gt/+^ ears observed in our analysis could also reflect changes in cell-cell adhesion or migratory properties that regulate the timing or speed of delamination of neuroblasts from the otic vesicle. One limitation of the single cell approach used in this study is the small number of genes that was used in the quantitative assay. Single cell RNA-Seq data analysis could provide a more comprehensive analysis and is most certainly needed to capture the full extent of gene expression changes in *Chd7*^Gt/+^ ears. In this respect, reduced expression of genes that promote retention of cells in the otic epithelium or increased expression of genes the promote delamination could also explain our findings. Genes involved in cell adhesion, epithelial-to-mesenchymal transitions, or effectors of other morphogenetic signaling pathways such as WNT, SHH, and BMP are potential candidates that warrant further study (for a review, see Wu and Kelley, 2012).

Our data suggest that expression of Notch signaling genes is altered in the ventral otocyst with loss of *Chd7*. Notch functions early in the pro-sensory cell differentiation pathway, and is critical for pro-neural (e.g., *Neurod1, Neurog1*) gene expression (i.e., fating prosensory cells to becoming neuroblasts vs. sensory epithelium) (Jeon et al., 2011). CHD7 has been demonstrated to regulate Notch gene family members, including *Hes5, Hey1, Jag1*, and *Notch3* (Engelen et al., 2011). Further studies are needed to help clarify whether CHD7 has a direct regulatory effect on Notch-related gene expression.

In summary, our results utilize three-dimensional reconstruction of the otocyst with single cell transcriptomic data (Durruthy-Durruthy et al., 2014, 2015) and show that this method produces consistent reconstruction of the organ even when gene expression is altered as a result of a complex genetic pathology. We demonstrate that loss of the chromatin remodeler *Chd7* leads to shifted identities of cells in the developing mouse otocyst toward neuroblast lineages. Our observations show that single cell analysis and spatial reconstruction of the developing inner ear can be applied to a mouse mutant of a human developmental disorder and establish several new lines of investigation for follow-up studies.

## Author Contributions

SH and DM conceived and designed the experiments. ES, RD-D, MB, LA, SH, and DM contributed reagents, materials, and analysis tools. RD-D, MB, and DM performed the experiments. ES, RD-D, SH, and DM analyzed the data and wrote the manuscript. RD-D generated the figures. All authors read and approved the manuscript for submission.

## Conflict of Interest Statement

The authors declare that the research was conducted in the absence of any commercial or financial relationships that could be construed as a potential conflict of interest.

## Supplementary Material

FIGURE S1 Single-cell qRT-PCR analysis of 466 cells from *Chd7* wild type and mutant otocysts. **(A)** Left and right ears from three *Chd7*^+/+^ (WT; E1, E6, and E8) and four *Chd7*^Gt/+^ (MUT; E3, E4, E5, and E7) embryos were microdissected and subjected to FAC sorting. Following separation, cells were individually placed into five 96-well plates (P1–P5), qRT-PCR was performed, and the data analyzed according to a quality control protocol. Following this, a total of 215 wild type and 251 *Chd7* heterozygous mutant cells remained for subsequent analyses. Principal component analysis was performed by embryo **(B)** and by plate **(C)**. Individual dots represent sorted cells, which occupy distinct groups in both analyses, ensuring lack of technical bias. Hierarchical clustering **(D)** also showed no differences attributable to distinct embryos or cell plates.

FIGURE S2 Principal component analysis of all cells for each embryo separately (wildtype, *n* = 3, mutant, *n* = 4). Each dot represents a cell and is projected onto the first two principal components. Cells are clustered by k-means and color-coded (red – putative neuroblast cells, blue = putative otic epithelial cells). Expression levels of two representative markers for both otic populations are shown. For both wildtype **(A)** and mutant **(B)** the ratio of neuroblast-associated cells and otic epithelial-associated cells was calculated and the difference was tested for statistical significance **(C)**.

TABLE S1 Gene-specific primer pairs used for the Fluidigm qPCR array.
