# Supplementary figures and images for: Single Cell Transcriptomics Reveal Abnormalities in Neurosensory Patterning of the Chd7 Mutant Mouse Ear
Source: Front Genet. 2018 Oct 23;9:473. doi: 10.3389/fgene.2018.00473 (PMC6232929; doi:10.3389/fgene.2018.00473)

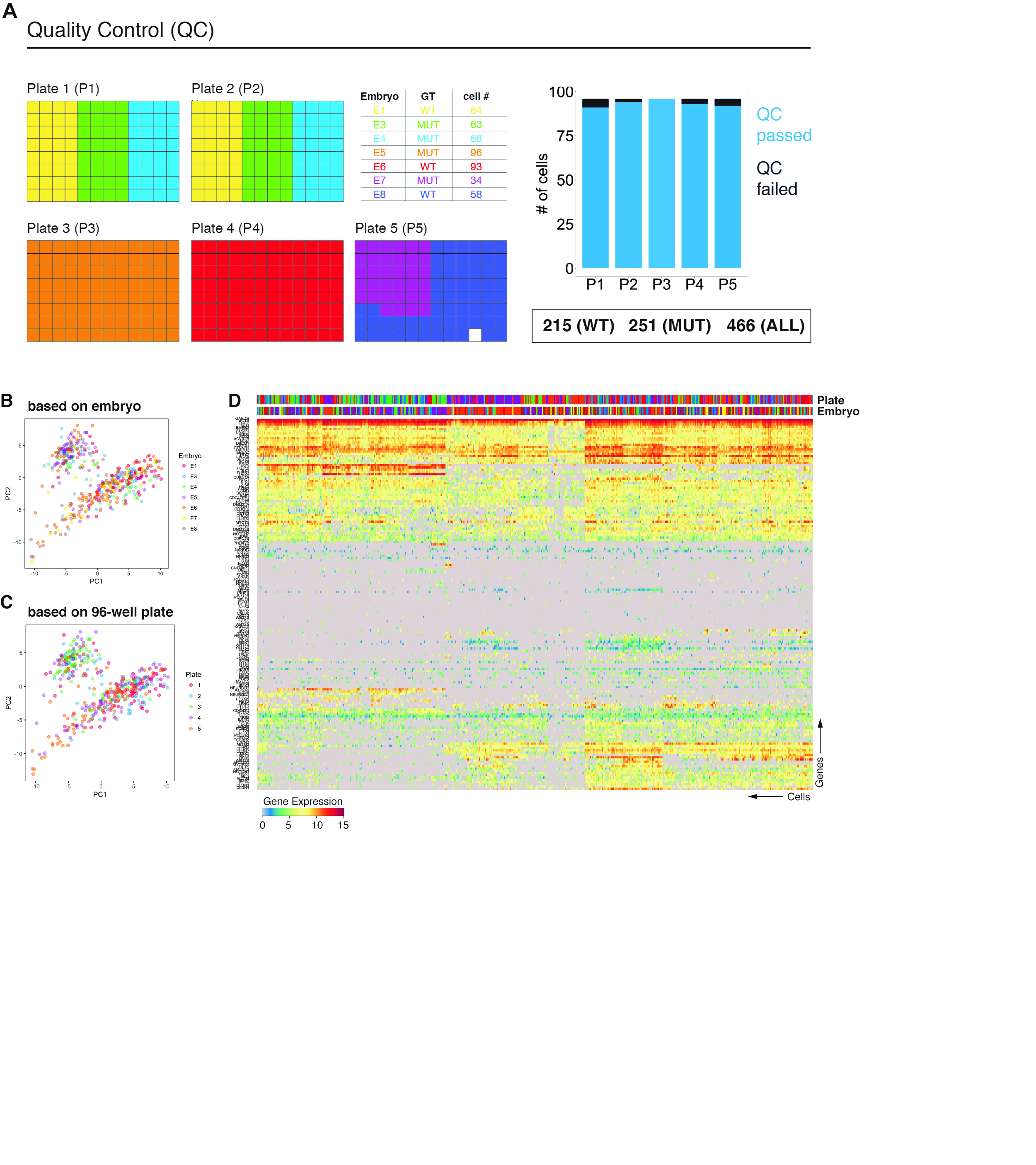

Supplement: FIGURE S1 — Single-cell qRT-PCR analysis of 466 cells from Chd7 wild type and mutant otocysts. (A) Left and right ears from three Chd7+/+ (WT; E1, E6, and E8) and four Chd7Gt/+ (MUT; E3, E4, E5, and E7) embryos were microdissected and subjected to FAC sorting. Following separation, cells were individually placed into five 96-well plates (P1–P5), qRT-PCR was performed, and the data analyzed according to a quality control protocol. Following this, a total of 215 wild type and 251 Chd7 heterozygous mutant cells remained for subsequent analyses. Principal component analysis was performed by embryo (B) and by plate (C). Individual dots represent sorted cells, which occupy distinct groups in both analyses, ensuring lack of technical bias. Hierarchical clustering (D) also showed no differences attributable to distinct embryos or cell plates. [file Image_1.TIF]

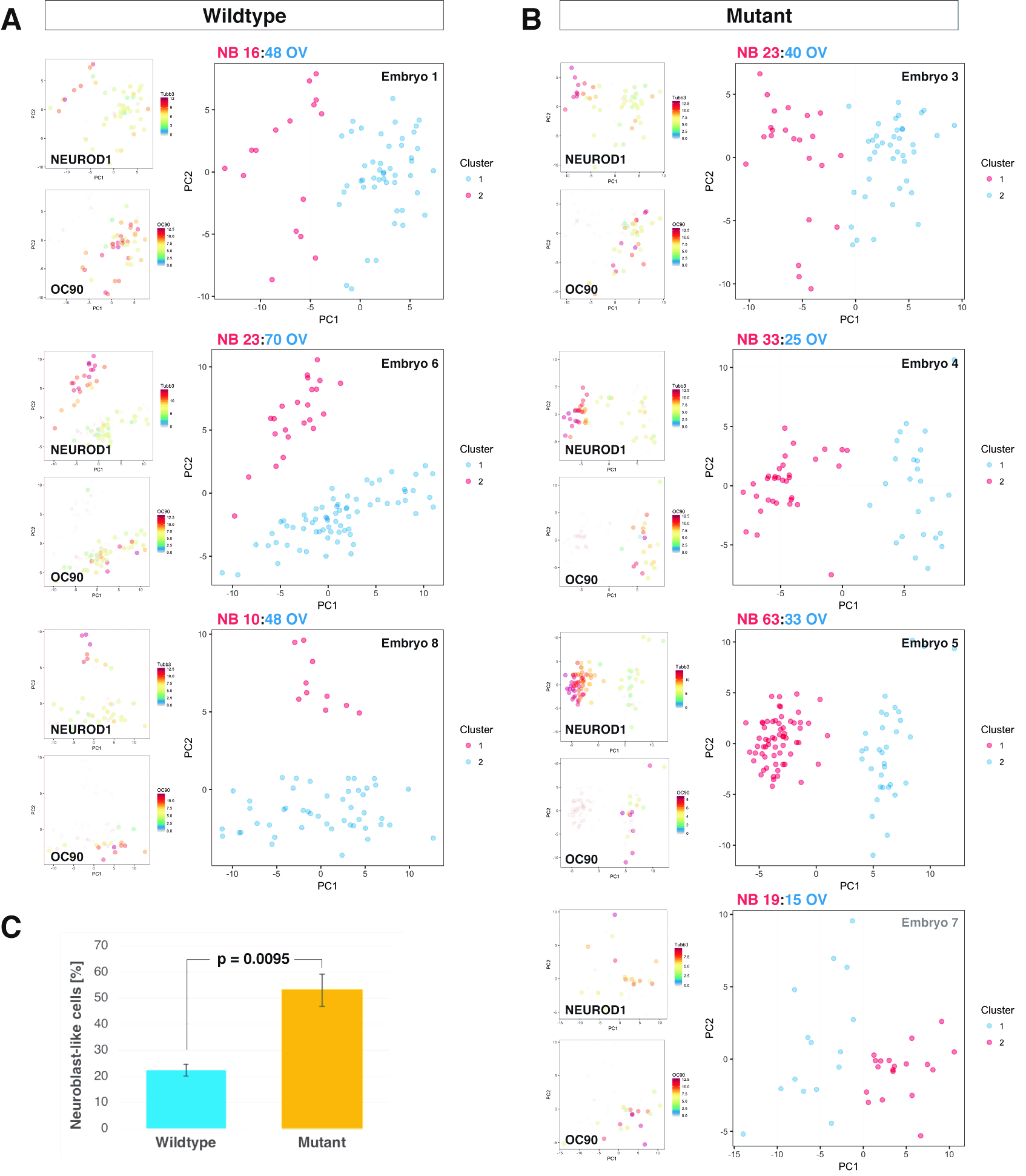

Supplement: FIGURE S2 — Principal component analysis of all cells for each embryo separately (wildtype, n = 3, mutant, n = 4). Each dot represents a cell and is projected onto the first two principal components. Cells are clustered by k-means and color-coded (red – putative neuroblast cells, blue = putative otic epithelial cells). Expression levels of two representative markers for both otic populations are shown. For both wildtype (A) and mutant (B) the ratio of neuroblast-associated cells and otic epithelial-associated cells was calculated and the difference was tested for statistical significance (C). [file Image_2.TIF]
