# Supplementary material for: Single Cell Transcriptomics Reveal Abnormalities in Neurosensory Patterning of the Chd7 Mutant Mouse Ear
Source: Front Genet. 2018 Oct 23;9:473. doi: 10.3389/fgene.2018.00473 (PMC6232929; doi:10.3389/fgene.2018.00473)
Supplement: TABLE S1 — Gene-specific primer pairs used for the Fluidigm qPCR array. [file Table_1.docx]

**Supplemental Table 1.** Gene-specific primer pairs used for the Fluidigm qPCR array.

| **Gene Symbol** | **Design RefSeq** | **Forward Primer** | **Reverse Primer** |
| --- | --- | --- | --- |
| *Acta2* | NM_007392.2 | GAGGCACCACTGAACCCTAA | TACATGGCGGGGACATTGAA |
| *Actb* | NM_007393.3 | CCCTAAGGCCAACCGTGAAA | CAGCCTGGATGGCTACGTAC |
| *Acvr2a* | NM_007396.4 | CCTACCCTCCTGTACTTGTTCC | ACAGCTGCAATGGCTTCAAC |
| *Acvr2b* | NM_007397.2 | GAACGACTTTGTGGCTGTGAA | CGGGTGTGCTGAAGATTTCC |
| *Afp* | NM_007423.4 | AAGCAAAGCTGCGCTCTCTA | GGGCTTTCCTCGTGTAACCA |
| *Ap1m2* | NM_001110300.1 | ATTGTGGGCACCATCAAGCT | AAAGAGCACTCGGTCATTGAGG |
| *Apcdd1* | NM_153000.4 | TCCTGCTCAGATACCTGTTCC | AGACCTGCTGTCTGGATGAA |
| *Atoh1* | NM_007500.4 | CCGTCCTTCAACAACGACAA | TCCGACAGAGCGTTGATGTA |
| *Atp2a1* | NM_007504.2 | TCACCACCAACCAGATGTCA | ACTCGTTCAGTGAGCAGACA |
| *Axin2* | NM_015732.4 | GATCCACGGAAACAGCTGAA | AGCCGGAACCTACGTGATAA |
| *Bdnf* | NM_007540.4 | TCCAAAGGCCAACTGAAGCA | CTGCAGCCTTCCTTGGTGTA |
| *Bmi1* | NM_007552.4 | AAACCAGACCACTCCTGAAC | TTTGAAAAGCCCTGGGACTAA |
| *Bmp2* | NM_007553.2 | GTGCGCAGCTTCCATCAC | CGTCACTGGGGACAGAACTTAA |
| *Bmp4* | NM_007554.2 | GAACCGGGCTTGAGTACCC | GGTCCCTGGGATGTTCTCC |
| *Bmp7* | NM_007557.2 | TGTACGTCAGCTTCCGAGAC | GTAGTAGGCAGCATAGCCTTCA |
| *Bmpr2* | NM_007561.3 | GGGGAAGAAGATAATGCGGCTA | GGTTCACAGCTCCTTCTAGCA |
| *Brip1* | NM_178309.1 | CTGCATTAGCATGGCAGCAA | AACATGAAGGTGGTGCCTCA |
| *Calb2* | NM_007586.1 | ACAGGCCCTATGATGAACCTA | AGACCCAATTTGCCATCTCC |
| *Cdc20* | NM_023223.2 | TCCCCTGCAAACATTCACTCA | ATATTGGACTGCCAGGGACAC |
| *Cdc42se1* | NM_172395.2 | ACAGACCGTGGAGCAATTCTA | TTGGGCTGGACACAGAATCA |
| *Cdh1* | NM_009864.2 | CAGATGATGATACCCGGGACAA | GTGCAGCTGGCTCAAATCAA |
| *Cdk1* | NM_007659.3 | TGCCAGAGCGTTTGGAATAC | CACTTCTGGAGATCGGTACCA |
| *Cdk7* | NM_009874.3 | GAGCGAAGCGCTATGAGAAA | TGTTCTTGTCCCTGGCCTTA |
| *Cdkn1a* | NM_007669.4 | GAACATCTCAGGGCCGAAAAC | TCTGCGCTTGGAGTGATAGAA |
| *Cdkn1b* | NM_009875.4 | CAGTGTCCAGGGATGAGGAA | TTCGGGGAACCGTCTGAAA |
| *Cdkn2a* | NM_001040654.1 | CCGACGGGCATAGCTTCA | GGGCTGAGGCCGGATTTA |
| *Cdkn2d* | NM_009878.3 | CTGAACCGCTTTGGCAAGAC | CCTTGCTTCAGGAGCTCCAA |
| *Chrd* | NM_009893.2 | CCTTTGGGGAGATGAGCTGTA | GAACAATCGTCCCGCTCAC |
| *Cldn10* | NM_001160096.1 | CAAAGTCGGAGGCTCAGATCAA | AATACAATCCCGGCCAAGCA |
| *Cldn3* | NM_009902.4 | GTGTACCAACTGCGTACAAGAC | GCCAACAGGAAAAGCACTCC |
| *Cldn4* | NM_009903.2 | CGTGGCAAGCATGCTGATTA | GGAAGCCACCATAGGGTTGTA |
| *Cldn6* | NM_018777.4 | GTCCGCAGACAAAGCTGAC | CGATCCCCAAGATTTGCAGAC |
| *Cldn7* | NM_016887.6 | CGGCATGATGAGCTGCAAAA | CACCAGGGACACCACCATTAA |
| *Cldn8* | NM_018778.3 | TTGCTGACAGCCGGAATCA | GAATTGGCAACCCAGCTGAC |
| *Clrn1* | NM_153384.2 | AAGTTCATGGGCGAGATGCA | CGGAAAGGCCTTGCTCCTAA |
| *Cntnap1* | NM_016782.2 | AATGTATCCATCGCGCAGAC | GTGCCAGAAGCCATCATTCA |
| *Ctnnb1* | NM_007614.3 | CATTGGTGCCCAGGGAGAA | GCCGTATCCACCAGAGTGAAA |
| *Dach1* | NM_007826.2 | TGACATGGGGCATGAGTCAAA | TCTTGCGGTTGGTGTGGAA |
| *Dach2* | NM_001142570.1 | TGGTGACAGTGGACTGAGAA | CGGCGTTCCACTGTTTTCTA |
| *Dkk1* | NM_010051.3 | CTATGAGGGCGGGAACAAGTA | TCATCTTCAGCGCAAGGGTA |
| *Dkk3* | NM_015814.2 | TGCCAGTTCTCCAGCTTCAA | TGGTCTCCACAGCACTCAC |
| *Dll1* | NM_007865.3 | TGGCTGGAAAGGCCAGTAC | CCCTGGTTTGTCACAGTATCCA |
| *Dlx5* | NM_010056.2 | TCTCTAGGACTGACGCAAAC | TGACTGTGGCGAGTTACAC |
| *Dlx6* | NM_010057.2 | GCTGCTTCCTTAGGACTGAC | TGGGTTACTACCCTGCTTCA |
| *Dner* | NM_152915.1 | ACATGTGTGTCCAGCCTCA | GGGTCCACCTTCTCTTCACA |
| *Dnmt1* | NM_010066.3 | AGCCATTGGCCTGGAGATTA | GCAGCCTCCTCTTTTGCTTTA |
| *Dnmt3a* | NM_007872.4 | CGCCAGAAGTGCAGAAACA | AATGAAGAGTGGGTGCTCCA |
| *Dnmt3b* | NM_001003961.3 | GACGTCCGGAAAATCACCAA | GATCATTGCATGGGCTTCCA |
| *eGFP* | eGFP.1 | TTCAAGGACGACGGCAACTA | TCAGCTCGATGCGGTTCA |
| *Egfr* | NM_207655.2 | CCTCCATGCTTTCGAGAACCTA | CCAACGACCGCCAAAGAAAA |
| *Emx2* | NM_010132.2 | CTCATCCACCGCTACCGATA | GAGCGTTGTGCAAAAGGAAAC |
| *En1* | NM_010133.2 | GGGTCTACTGCACACGCTAT | CGCTTGTCTTCCTTCTCGTTC |
| *Epcam* | NM_008532.2 | GGTGAATGCCAGTGTACTTCC | CTGCTTTCATCGCCAAGCA |
| *Epha4* | NM_007936.3 | ACTCATCCGCAATCCCAACA | ATTCAGGGGAGCTGGGATCTA |
| *Epha7* | NM_010141.3 | GGAGCATCCCAATGGAGTCA | AGTGTTGAGTACGTCCTTTCCC |
| *Ephb3* | NM_010143.1 | CATGGACACGAAATGGGTGAC | TGGCTTCATCATAGCCGCTTA |
| *Espn* | NM_019585.3 | GCAGAAGATGCAGGAGGAA | CGAAGAATGTCTCGTCTCCA |
| *Etv4* | NM_008815.2 | CGCTCGCTGCGATACTATTA | GCTCGCACACAAACTTGTAC |
| *Eya1* | NM_010164.2 | CAGCAGACGGGTCTTTAGACA | GGTGAGCTGGTCTTGGACTAAA |
| *Eya2* | NM_010165.2 | GCTCTATCAAGGCGCCAAC | AGCTGGGGTAGGACGGATA |
| *Fbxo2* | NM_176848.1 | GGACCACTGGCAACAGTTCTA | TCGCTCCAGCCCTCCAA |
| *Fgf10* | NM_008002.4 | GCGGGACCAAGAATGAAGAC | GTTGCTGTTGATGGCTTTGAC |
| *Fgf3* | NM_008007.2 | TGGCCATGAACAAGAGAGGAC | TGGATCCGTTCCACAAACTCA |
| *Fgf4* | NM_010202.5 | TGGTGTGCACGCAGACAC | GCCACTCCGAAGATGCTCAC |
| *Fgf8* | NM_010205.2 | CATCAACGCCATGGCAGAA | ACTCGGACTCTGCTTCCAAA |
| *Fgf9* | NM_013518.4 | ACGGTACTATCCAGGGAACCA | GACCAGGCCCACTGCTATAC |
| *Fgfr1* | NM_001079908.1 | GAGTAAGATCGGGCCAGACA | TCCATTTCCTTGTCGGTGGTA |
| *Fgfr2* | NM_010207.2 | TCAAGTGGATGGCTCCTGAA | CACATTAACACCCCGAAGGAC |
| *Fgfr3* | NM_008010.4 | AGGATTTAGACCGCATCCTCAC | CCTGGCGAGTACTGCTCAAA |
| *Fgfr4* | NM_008011.2 | CTTCCACGGGGAGAATCGTA | CGAGGGTACCACACTTTCCA |
| *Foxa2* | NM_010446.2 | GGGAGCCGTGAAGATGGAA | TCATGTTGCTCACGGAAGAGTA |
| *Foxg1* | NM_001160112.1 | GCCAGCAGCACTTTGAGTTA | TGAGTCAACACGGAGCTGTA |
| *Foxi2* | NM_207426.2 | CTTTCTACAAGCGCAGCAA | CGGGGCACCTTCTTGAA |
| *Foxi3* | NM_001101464.1 | CCGCCACAACCTATCACTCA | GGATCCAGAGTCCAGTAATTACCC |
| *Fst* | NM_008046.2 | AGCAGCCGGAACTAGAAGTAC | TGGAGCTGCCTGGACAAAA |
| *Fzd3* | NM_021458.2 | TGACCAACAGACTGCAGCTTTA | ATGGCCGAAAATCCCGAGAA |
| *Fzd4* | NM_008055.4 | CTGCAGCTGACAACTTTCAC | GTGCACATTGGCACATAAACC |
| *Fzd6* | NM_008056.3 | CGATGGCCTGAAGAACTTGAA | AGCTCTGTGTGTGGATGAGAA |
| *Galnt3* | NM_015736.2 | TGGAAATCTGGGGAGGTGAA | AGAGCAAGGCATAATCTCCAAC |
| *Gapdh* | NM_008084.2 | AGACGGCCGCATCTTCTT | TTCACACCGACCTTCACCAT |
| *Gata2* | NM_008090.5 | CACCCCTAAGCAGAGAAGCAA | TGTGGCACCACAGTTGACA |
| *Gata3* | NM_008091.3 | CCTACCGGGTTCGGATGTAA | CCGCAGTTCACACACTCC |
| *Gbx2* | NM_010262.3 | CAAGCGGATGCGGAAGAC | CCCTTCGGGTCATCTTCCA |
| *Gdf10* | NM_145741.2 | CCCAAATCCTTTGACGCCTAC | CAATGCCCACAGCTCTGAC |
| *Gfap* | NM_010277.3 | AGAACAACCTGGCTGCGTATA | CAGCGATTCAACCTTTCTCTCC |
| *Gli1* | NM_010296.2 | CAGAATCGGACCCACTCCAA | GCGAGCTGGGATCTGTGTA |
| *Gli2* | NM_001081125.1 | ACCAGGAGGGAAGGTACCATTA | GATGACAGGGCTGCCACTTA |
| *Gli3* | NM_008130.2 | CCGTAGCAGCTCTTCAGCAA | GGGTAGGTGAAGCTCAATGCA |
| *Gpr98* | NM_054053.4 | GAACCCTCAGCCACATACAA | TCACTGTGATCTCCCCTGAA |
| *Hand2* | NM_010402.4 | GACCGACGTGAAAGAGGAGAA | TTTCTTGTCGTTGCTGCTCAC |
| *Hapln1* | NM_013500.4 | CAGCTACACTCCTCCAGATCA | CTTGGCTTGTTCTGCTTCCA |
| *Has2* | NM_008216.3 | GGGGTGGAAAGAGAGAAGTCA | ATGAGGCAGGGTCAAGCATA |
| *Hes1* | NM_008235.2 | TGAAGCACCTCCGGAACC | CGCGGTATTTCCCCAACAC |
| *Hes2* | NM_008236.4 | GAGAGCCTAAGCCAGCTGAA | AGGATGTCTGCCTTCTCCAAC |
| *Hes5* | NM_010419.4 | AAGAGCCTGCACCAGGACTA | GTGCAGGGTCAGGAACTGTAC |
| *Hes6* | NM_019479.3 | CCGAGGTTCTGGAGCTGAC | AAGCGCTCGCTTGCTTCA |
| *Hey1* | NM_010423.2 | ACGAGACCATCGAGGTGGAA | CGTTGGGGACATGGAACACA |
| *Hey2* | NM_013904.1 | GTGGGGAGCGAGAACAATTAC | TGTCGGTGAATTGGACCTCA |
| *Hmx2* | NM_145998.3 | AAGCCCTAAGCACCATACCC | CGGGTGAGAAGGAGAAAGGAA |
| *Hmx3* | NM_008257.3 | GCCTGGTGGTACCCCTACA | CGCAGGAGGGCCTTTTCC |
| *Hoxa1* | NM_010449.4 | TCTTCTCCAGCGCAGACC | ACGTAGCCGTACTCTCCAAC |
| *Hoxa3* | NM_010452.3 | AGTCAAGGCAGAACACTAAGCA | CAGGCGGGCTCTTGTCA |
| *Hoxb1* | NM_008266.5 | GAAGGTCAAGAGAAACCCACCTA | GTGAAGTTTGTGCGGAGACC |
| *Hoxd3* | NM_010468.2 | AAGAATCCCGACAGAACTCCA | GTGGGCTCTTGTCCTCACA |
| *Inhba* | NM_008380.1 | GCAGACCTCGGAGATCATCA | GTCACTGCCTTCCTTGGAAA |
| *Irx1* | NM_010573.2 | CAGCGCCTTCTTGCCCTA | CGGGGTTGTCCTTCAGTTCA |
| *Irx4* | NM_018885.2 | TCCTACCCGCAGTTTGGATA | AGGGAGTTGGTGGTCATCA |
| *Isl1* | NM_021459.4 | GGACAAGAAACGCAGCATCA | GTTCCTGTCATCCCCTGGATA |
| *Jag1* | NM_013822.4 | TCCCAAGCATGGGTCTTGTA | GATGCACTTGTCGCAGTACA |
| *Jag2* | NM_010588.2 | CTCGTCGTCATTCCCTTTCA | GGTGTCATTGTCCCAGTCC |
| *Kcnj10* | NM_001039484.1 | TATCAGAGCAGCCACTTCAC | CGTATTCCTGGGGCCACTA |
| *Kcnq4* | NM_001081142.1 | ACAGCCACCTGGTATTACTATGAC | GCCCGTTGTATGTGCTCAAA |
| *Krt14* | NM_016958.1 | TGGCTGCCGATGACTTCC | CCATTGATGTCGGCCTCCA |
| *Lfng* | NM_008494.3 | TCGATCTGCTGTTCGAGACC | CCTCCCCATCAGTGAAGATGAA |
| *Lgr5* | NM_010195.2 | CTCCAACCTCAGCGTCTTCA | ATGTAGGAGACTGGCGGGTA |
| *Lhx1* | NM_008498.2 | ACGCCATATCCGTGAGCAA | TTGGAGCGTCGATTCTGGAA |
| *Lhx2* | NM_010710.3 | AAAAGACCGGCCTCACCAA | CGTAAAAGGTTGCGCCTGAA |
| *Lin28a* | NM_145833.1 | ACATGCAGAAGCGAAGATCC | CTTGGCATGATGGTCTAGCC |
| *Lmx1a* | NM_033652.5 | CAACAGCAACAGGACCAACA | ATGCCCGCATTCCCACTA |
| *Mbd6* | NM_033072.2 | TGCGAGAAGGTGCTGTGTA | TAGCTACGGGTTTGCTCCAA |
| *Mcam* | NM_023061.2 | AGTGGGAACCACATGAAGGAA | GCTCTACCTCCACCCACAC |
| *Msi2* | NM_001201341.1 | TGAGAGATCCCACAACGAAAC | ATGGTGGGGCTGACCTAATA |
| *Msx1* | NM_010835.2 | AAAGCCCCGAGAAACTAGATCG | GCTTGCGGTTGGTCTTGTG |
| *Mycn* | NM_008709.3 | CGTAAGCGCGCACACAC | GTCGCGCCAACCTCCAA |
| *Myo7a* | NM_008663.2 | CTTCTGGCTGCCATCCTACA | AGGACTTCACACGCATCCAA |
| *Nab2* | NM_001122895.1 | TCTTCTCACTGTCCCGACAA | TGGGCCTCCCAATTCTTCA |
| *Nanog* | NM_028016.2 | TCTGGGAACGCCTCATCAA | GAGGCAGGTCTTCAGAGGAA |
| *Nes* | NM_016701.3 | AGCAACTGGCACACCTCA | TCTGCAAGCGAGAGTTCTCA |
| *Neurod1* | NM_010894.2 | CCAGGGTTATGAGATCGTCAC | TCGTCCTGAGAACTGAGACA |
| *Neurog1* | NM_010896.2 | ACTCTCTGACCCCAGTAGTCC | GCAGGCCAGGAAAGGAGAAA |
| *Neurog2* | NM_009718.2 | GACATTCCCGGACACACAC | CTCCTTCAACTCCAGAGTCTCA |
| *Neurog3* | NM_009719.6 | TGACCTGCTGCTCTCTATTCT | TGTGTCTCTGGGGACACTTG |
| *Nog* | NM_008711.2 | AGCAAGAAGCTGAGGAGGAA | TAGGTCATTCCACGCGTACA |
| *Notch1* | NM_008714.3 | GGACGGCGTGAATACCTACA | GACATTCGTCCACATCCTCTGTA |
| *Notch2* | NM_010928.2 | TGGTTCTGGGACAAGTGAACA | ACAGCAAAGCCTCATCCTCA |
| *Notch3* | NM_008716.2 | CCATGCCGATGTCAATGCA | TAGCCTCCACGTTGTTCACA |
| *Npnt* | NM_033525.2 | GATACATGCTGCTGCCAGAC | ACGTCACAGCCATACTGACA |
| *Ntf3* | NM_001164034.1 | CCGAGCACTGACTTCAGAAAAC | TTCTCGACAAGGCACACACA |
| *Oc90* | NM_010953.2 | CAGATGTGGATTGCACCAACAA | AGCCTTGTCACACGTACACA |
| *Otol1* | NM_001018031.2 | GTTCATTTGTCACTGGGCAGAA | CCAGTGTCACCAGTCTCTCC |
| *Otx1* | NM_011023.3 | AAGATCAACCTGCCAGAGTCC | CGGGTTTTCGTTCCATTCCC |
| *Otx2* | NM_144841.3 | GTATGGACTTGCTGCATCCC | TCGAGCTGTGCCCTAGTAAA |
| *Pax2* | NM_011037.3 | CCATGGCTGTGTCAGCAAAA | GCTTGGAGCCACCAATCAC |
| *Pax3* | NM_001159520.1 | TTACCAGCCCACGTCTATTCC | GTGTACAGTGCTCGGAGGAA |
| *Pax5* | NM_008782.2 | GCCACCCTCAGTATTCTTCCTAC | GGCTGCAGGGCTGTAATAGTA |
| *Pax6* | NM_013627.4 | TATCCCGGGACTTCAGTACCA | TGATGGAGTTGGTGTTCTCTCC |
| *Pax8* | NM_011040.3 | CAGCCTGGCAATGACAACAA | TGCTCTGTGAGTCGATGCTTA |
| *Pcdh15* | NM_023370.2 | ACTGCTGTGGACCAGGATAA | GGGCAAAGATGCTGTTGGTA |
| *Phox2b* | NM_008888.3 | AGCACTGAAGATCGACCTCAC | CCTGCTTGCGAAACTTAGCC |
| *Pou3f3* | NM_008900.2 | CGCATCAAGCTGGGTTTCAC | TGCGAGAACACGTTGCCATA |
| *Pou4f3* | NM_138945.2 | ATGCGCCGAGTTTGTCTCC | GCCAGCAGGCTCTCATCAAA |
| *Pou5f1* | NM_013633.2 | TCCCTACAGCAGATCACTCAC | CGCCGGTTACAGAACCATAC |
| *Prox1* | NM_008937.2 | GCCCTCAACATGCACTACAAC | CGTGATCTGCGCAACTTCC |
| *Ptch1* | NM_008957.2 | TGCTGGAGGAGAACAAGCAA | CCAGTCACTGTCAAATGCATCC |
| *Ptch2* | NM_008958.2 | GTGGCTGTAATTGAGACAGACC | CCCAGCTTCTCCTTGGTGTA |
| *Rarb* | NM_011243.1 | ACAGATCTCCGCAGCATCA | AGGTGGCATTGATCCAGGAA |
| *Rplp0* | NM_007475.5 | CCGCCTGGTTCTCCTATAAAA | CCACGCGGGGTTTAAAGAC |
| *Shh* | NM_009170.3 | AGGAAAACACGGGAGCAGAC | ACAGAGATGGCCAAGGCATTTA |
| *Six1* | NM_009189.3 | AAGAACCGGAGGCAAAGAGA | CCCTTCCAGAGGAGAGAGTTG |
| *Six3* | NM_011381.4 | GGTTTAAGAACCGGCGACAG | CCCGATGGCCTGATGCT |
| *Six4* | NM_011382.2 | GGGAGCATTGGATTCTCTCCA | GTCCGAAGTGCTTGGGGTA |
| *Slc26a9* | NM_177243.4 | AGAAGAGGAAGGTCCCAGCTA | CAGTCAGGGTCTCTGTGTGAAA |
| *Smo* | NM_176996.4 | AGGCAGCCAGCAAGATCAA | GGTGATGAGCACAAAGCCAAA |
| *Snai1* | NM_011427.2 | CGCCGGAAGCCCAACTATA | AGGTGGGCCTGGTCGTA |
| *Snai2* | NM_011415.2 | CAGACCCTGGCTGCTTCA | TTGCAGTGAGGGCAAGAGAA |
| *Sox10* | NM_011437.1 | ACCAGTACCCTCACCTCCA | GCGCTTGTCACTTTCGTTCA |
| *Sox17* | NM_011441.4 | AACCTCAGCATGTCACCTCA | ACCTCTTGGGGAAATAGGAAGG |
| *Sox2* | NM_011443.3 | TGAAGGAGCACCCGGATTATA | CGGGAAGCGTGTACTTATCC |
| *Sox3* | NM_009237.2 | GAGAACCCCAAGATGCACAAC | GCATCGGTCAGCAGTTTCC |
| *Sox9* | NM_011448.4 | AGTACCCGCATCTGCACAA | GTCTCTTCTCGCTCTCGTTCA |
| *Spp1* | NM_009263.2 | AATGCTGTGTCCTCTGAAGAAAA | CCATGTGGTCATGGCTTTCA |
| *Spry1* | NM_011896.2 | ACTGCACCAAGACCCGAAAA | GGTGCTCGTAGCTGCTATTCA |
| *Spry2* | NM_011897.3 | GGAGAGGGGTTGGTGCAAA | AGGTCTTGGCAGTGTGTTCA |
| *T* | NM_009309.2 | ATGCTGCCTGTGAGTCATAAC | GGTACCATTGCTCACAGACC |
| *Tbx1* | NM_011532.1 | ACCGAGATGATCGTCACCAA | GCCATGGGATCCATTCCAAAA |
| *Tbx2* | NM_009324.2 | CTTCCCAGAGACCGACTTCA | AGGGGTTGTTGTCGATCTTCA |
| *Tgfb3* | NM_009368.3 | TCAGGCCCTTGCCCATAC | CTCTGGGTTCAGGGTGTTGTA |
| *Tgfbr2* | NM_009371.3 | TCTGTGAGAAGCCGCATGAA | GGCAAACCGTCTCCAGAGTAA |
| *Trp63* | NM_001127259.1 | GAGCGTGCTGGTCCCTTA | GCAGCTGCTGTTACACATGAA |
| *Trpm3* | NM_001035239.2 | AAGCAGCCATGACAACTGGA | GGCATCTCCAACATGACGAATG |
| *Tubb3* | NM_023279.2 | GCGCATCAGCGTATACTACA | AGGTTCCAAGTCCACCAGAA |
| *Vax1* | NM_009501.1 | TCCTCAGCGGACCCAGATTA | GGGCAGGATGATTTCTCGGATA |
| *Vax2* | NM_011912.3 | CGCTCGGTGAGACAGACC | CCTTGGGCAAGACAATTTCCC |
| *Wnt1* | NM_021279.4 | CTCTTCGGCAAGATCGTCAAC | CGCCACGGAATGTGTGAC |
| *Wnt2b* | NM_009520.3 | GGGCCCTCATGAACTTACACA | ACACTTGCACTCCAGCTTCA |
| *Wnt3* | NM_009521.2 | CGCTCAGCTATGAACAAGCA | GACAACCCGTGGCATTTACA |
| *Wnt3a* | NM_009522.2 | CTGCCATGAACCGTCACAAC | GCACTTGAGGTGCATGTGAC |
| *Wnt4* | NM_009523.1 | TCTTTGGGAAGGTGGTGACA | CATGCCCTTGTCACTGCAAA |
| *Wnt6* | NM_009526.3 | GGTGCAACTGCACAACAAC | GAAAGCCCATGGCACTTACA |
| *Wnt7a* | NM_009527.3 | CCCGGACGCTCATGAACTTA | GGCACTTACACTCCAGCTTCA |
| *Wnt7b* | NM_009528.3 | CGCCTCATGAACCTTCACAAC | CCTGACACACCGTGACACTTA |
| *Wnt8a* | NM_009290.2 | CCTGGTGAACCTTCACAACAA | GCTTCCTGAGATGCCATGAC |
